# Cases Extracted from the Note-Book of Henry Davies, M.D., Physician to the British Lying-In Hospital, &c.

**Published:** 1834-07-01

**Authors:** 


### ORIGINAL COMMUNICATIONS.

*Cases extracted from the Note-book of* HENRY DAVIES, M.D.,
Physician to the British Lying-in Hospital, &c.

### CASE I. *Marasmus.*

OCTOBER 4th, 1820. Frederic Thomson, æt. seventeen months, has been wasting away for some weeks. His stools are slimy, and come away in jerks; he has three or four in quick succession, and then none for perhaps half a day. His skin is flabby and wrinkled; his belly hard, but not tumid. The patient moans much, and is very fretful. His appetite is bad, and he loathes everything except potatoes, which he will eat voraciously, and which are discharged undigested. He has four incisor and four molar teeth. He sleeps well, and indeed is always torpid.—℞. Hydr. Submur.; Ipecac. pulv. āā gr. j. M. ut ft. pulv. omni nocte sumend.—℞. Magn. Sulph. ʒij.; Aquæ Carui ℥ss; Aquæ fontanæ ʒx.; Syrupi simpl. ʒij. M. sumat quartam partem bis in die.

The patient was ordered to use the tepid salt-water bath; and a stimulant embrocation was applied to the abdomen, which was also supported by a roller.

16th. A mixture of Inf. Cascarillæ and Magn. Sulph. was substituted for the previous one: the powder, bath, and embrocation were continued.

23d. An enema of mutton-broth was ordered to be injected every night; the other medicines to be continued.

25th. The patient is better.

30th. The stools are more natural in consistence, and he has two a day only: he takes his food well.—Continuentur Pulv. et Mist. Cascar. et enema.

November 6th. The belly is natural, the appetite good, and he has only three stools a day, which are of healthy appearance. The child is no longer so torpid, but he has a troublesome cough. He has cut another incisor. He was ordered to take an emetic immediately, and continue the cascarilla and the enema.

December 16th. Haust. Sennæ p.r.n. Rept. Cascar., &c.

27th. Belly regular; appetite good, sleep undisturbed. He still has a little cough, with a pale face and puffy skin. Sumat Vin. Ferri gtt. xv. ter in die. Rep. Haust. Sennæ p.r.n.

January 10th, 1821. The cough has left him. Contr. Vin. Ferri, &c.

31st. Discharged cured.

Case ii. *Marasmus, with Omental Tumour.*

1821. — Frame, æt. nine, had been attacked, about twelve months before, with pain in the left side and umbilical region, for which a blister and medicine were prescribed. The patient has since been under the care of various practitioners, but without any permanent benefit. The mother says that she has lost four children by similar diseases; the three now living are remarkably well-looking, with light eyes, and a fair delicate skin. The patient died July 21st, and the following post-mortem appearances were found on the 23d.

The abdomen was very tumid, and its parietes were green, as also were those of the thorax. On laying open the abdomen, the stench was intolerable, and a very large quantity of sero-purulent fluid was discharged. The intestines were coated with a deposition of coagulum, and were generally adherent to each other. The peritoneal lining of the parietes was in a state of suppuration, and resembled dirty fat mottled with spots of charcoal, while to the touch it had a granulated feel. The omentum was drawn under the left hypochondrium, covering most part of the stomach and transverse arch of the colon; to both of which it was adherent. It was spotted with black granulations, broke easily between the fingers, and looked like anything else as much as omentum. When the small intestines were cleared of a portion of the pustular deposition, red vascular patches were seen upon them, and their mucous coat was in several places destroyed by oval ulcerations. The colon was contracted, and the stomach smaller than usual. The mesenteric glands were very much enlarged, probably weighing about a pound; and one of them, close to the vertebræ, was in a state of suppuration.

Case iii. *Abscess in the Groin, from Swallowing a Pin.*

May, 1824. John Batt, æt. twenty-three months. When seven weeks old, a pin slipped into his mouth, as his mother was dressing him; two months after which a swelling was observed in the right groin. When the child was a year old the swelling increased, and was situated a little above the internal ring. It was reduced by leeches and poultices, but returned repeatedly, and was removed by the same means. At the age of fourteen months it re-appeared, and, as it did not yield to the usual remedies, and presented an evident fluctuation, an opening was made in it, and a considerable quantity of very fetid pus was evacuated. Fæcal matter was observed on the poultices about two months after making the incision.

When the patient was twenty-three months old he was attacked with measles, of which he died.

*Sectio Cadaveris.* A probe being introduced at the orifice, and cut upon, a sinus was found, extending an inch and a half in length above the opening; below this there was a small inguinal hernia, with an orifice in the gut, where a black substance was observed. This proved to be a corroded pin, with its point turned outwards.

Case iv. *Encysted Omental Dropsy.*

December 12th, 1825. G. Upton, æt. two years and eight months. When two years old, his belly began to swell, and the urine was much reduced in quantity, and became of a milky colour. The abdomen is now greatly enlarged, tense, and protuberant, and the superficial veins are considerably distended. There are generally two motions daily, which are of a dark colour. The patient is very restless during the night, and screams occasionally in his sleep. He has cut all his teeth, and is able to walk about a little. He has been under the care of different practitioners for several months. ℞. Hydr. Submur.; Scillæ pulv.; Digital. fol. pulv. āā grss. M. ut ft. pulv. omni manè and nocte sumendus. Frictions to the abdomen were likewise ordered, and a flannel bandage.

16th. The abdomen has not diminished in size; there is no increase of urine, and the stools are still dark and slimy; on the other hand, his appetite was improved, and he sleeps better. Pulse 112.—Pergat.

23d. As the tension of the abdomen was extreme, and the patient appeared in great suffering, he was tapped to-day midway between the anterior and superior spinous process of the ilium and the linea alba, and nine pints of a greenish fluid were drawn off. He bore the operation extremely well, and was not sick during or after the evacuation of the water, but appeared much relieved by being freed from the pressure of so large a quantity of fluid.

25th. He has been very lively since the operation, and has slept well.

January 6th, 1826. The fluid appears to be collecting again. Small doses of elaterium were given, and with temporary benefit, as they increased the quantity of urine.

12th. The abdomen is tense, and the patient very restless.

18th. Paracentesis was performed to-day in the linea alba, midway between the symphysis pubis and umbilicus, and about four pints of fluid were drawn off. For three days after the operation the patient went on well, but afterwards became very restless. There was great thirst, accompanied by retching, so that whatever was taken was soon afterwards rejected. Small doses of calomel and pulvis antimonialis allayed the vomiting considerably.

On the 25th he was evidently sinking; and died on the 27th.

*Post-mortem Examination.* On opening the abdomen, a distinct sac was observed, which adhered firmly to the peritoneum, and was found, on separating it from its connexions, to be the great omentum, the layers of which were thickened, so that the parietes of the sac might be about a quarter of an inch in thickness: its colour was a pale yellow, with a slight blush of redness throughout its substance. This cavity contained a considerable quantity of purulent matter mixed with a little serum.

Under the great lobe of the liver there was a thin semi-transparent cyst, containing about a pint of thin watery fluid. The other abdominal viscera were sound. A small quantity of water was found in the ventricles of the brain. The left eye exhibited an excellent specimen of fungus hæmatodes in its early stage; but the optic nerve, at its entrance into the orbit, did not present any trace of disease.

